# Chlordiazepoxide-induced delirium in a patient undergoing alcohol withdrawal: a case report

**DOI:** 10.1186/s13256-022-03456-x

**Published:** 2022-07-07

**Authors:** Melissa A. Arabadjief, Omar H. Elsayed, Sabina Bashir, Meenakshi R. Gundumalla, Derek S. Menefee, Cody L. Bergman, Nayeem Z. Moulana, Rif S. El-Mallakh

**Affiliations:** 1 Department of Psychiatry and Behavioral Sciences, University of Louisville School of Medicine, Louisville, USA; 2 Department of Psychiatry and Behavioral Sciences, University of Louisville School of Medicine, 401 East Chestnut Street, Suite 610, Louisville, KY 40241 USA

**Keywords:** Acetylcholine, Alcohol withdrawal, Benzodiazepine, Case report, Chlordiazepoxide, Delirium tremens, Ethanol withdrawal

## Abstract

**Background:**

Ethanol dependence is associated with a discontinuation withdrawal delirium. Chlordiazepoxide is frequently successfully used in its treatment.

**Case presentation:**

A 27-year-old, Caucasian female with ethanol dependence who had objective symptoms of withdrawal experienced worsening of her delirium after administration of chlordiazepoxide, but improved with lorazepam and cleared with discontinuation of benzodiazepine administration.

**Conclusions:**

Worsening of delirium appears to be related to the specific use of chlordiazepoxide, but the mechanism of this effect is not clear. While this case does not alter the standard care of ethanol dependence, it does alert clinicians that our treatment approach may not be fully benign.

## Introduction

Delirium is a potentially reversible but life-threatening acute brain syndrome characterized by altered consciousness and short-term confusion, along with disturbances in cognition. Ethanol withdrawal is a well-established etiology of delirium (ethanol withdrawal delirium, EWD) in hospital settings and is commonly referred to as delirium tremens (DTs). EWD occurs in 0.2–0.7% among the general population and in as many as 5–12% among those in inpatient treatment for ethanol dependence or withdrawal [1–4]. Mortality with untreated acute EWD is around 20%, but the risk may also be increased beyond the acute phase. In a Finnish study, 37% of patients with EWD died over an 8-year follow-up period, with a hazard ratio (HR) of 20 compared with those without ethanol dependence and an HR of 12 compared with study participants with ethanol dependence but not EWD [4].

Benzodiazepines (BNZs) are well established as a first-line treatment for prevention and treatment of ethanol-related delirium [5]. However, BNZs are an independent risk factor for the exacerbation of delirium in severely ill patients not undergoing ethanol withdrawal [5], and may induce delirium with excessive use [6]. It has been proposed that the use of symptom-triggered administration of benzodiazepines, such as with the Clinical Institute Withdrawal Assessment for Alcohol-Revised (CIWA-Ar) [7], would reduce the likelihood of this complication of the aggressive treatment of EWD [8]. But CIWA-Ar has significant subjectiveness, which may reduce the utility of this approach, and control groups may even do better in some studies [9]. Recently, Wang *et al.* presented four cases of iatrogenic delirium secondary to sustained or increased dosing with BNZs due to use of CIWA-Ar in patients with histories suggestive of recent ethanol cessation and at risk of EWD [10]. However, herein, we report a case of BNZ-induced delirium in a patient receiving a relatively small dose of chlordiazepoxide in response to clear objective signs of alcohol withdrawal.

## Case presentation

A 27-year-old, Caucasian female with a past medical history of tachycardia and ethanol dependence presented to the emergency department (ED) for possible psychosis in the setting of ethanol withdrawal. Two days previously, the patient was admitted to an outside hospital for management of ethanol withdrawal and was treated with intravenous fluids but not BNZs. She removed her IV and left the hospital before being formally discharged, but the family brought her to our hospital with a report of paranoia and believing people outside her home were trying to kill her. Her last known ethanol consumption was 4 days prior, and alcohol, BNZs, and barbiturates were not detected in her serum or urine. She reported at least five “heavy pours” of vodka every day, and had consumed this amount for a few years. Examination was significant for tachycardia (127–132 beats per minute), high blood pressure (ranging from 143 to 149/102 to 103 mmHg), and mild tenderness to palpation to bilateral upper abdominal quadrants. She was calm, fully oriented, and with organized thought process, but endorsed paranoia regarding family members being out to get her and hearing voices outside her head talking about her. A medical workup for delirium revealed normal complete blood count, a comprehensive metabolic panel with mildly elevated transaminases [aspartate aminotransferase (AST) 94 U/L; alanine transaminase (ALT) 72 U/L], and a noncontrast computed tomogram of the brain with mild diffuse volume loss greater than expected for her age. These abnormalities were consistent with her drinking history and not indicative of a separate process. No substances were present in urine or serum toxicology. She was negative for coronavirus (COVID), human immunodeficiency virus (HIV), and hepatitis panel. She expressed interest in achieving sobriety from ethanol. She was first given lorazepam 2 mg orally and started on chlordiazepoxide 25 mg twice daily because her pulse and blood pressure remained elevated. Within 2 hours of every chlordiazepoxide dose, she became acutely confused and agitated, reporting visual and auditory hallucinations and stating that there were people on the other side of the door who were coming after her. After two doses of chlordiazepoxide (total of 50 mg, which is equivalent to 2 mg lorazepam), it was discontinued and she was treated with 2 mg of intramuscular lorazepam and a subsequent dose of oral lorazepam 2 mg, which were associated with improvement of her agitation. All BNZs were subsequently held. The next day, the patient was calm, coherent, with organized thought, and no hallucinations or paranoia. She remained that way over an extended observation period of over 10 hours. She was then referred to a substance treatment program where she did not exhibit any psychosis, and did not require additional BNZ medication.

Our institutional human subjects protection program allowed submission of this case presentation (IRB number 22.1081). Additionally, written informed consent was obtained from the patient for publication of this case report.

## Discussion

In this case, there is temporal association between administration of chlordiazepoxide to a nonelderly patient with objective autonomic symptoms of severe ethanol withdrawal and worsening of psychosis at the time of maximal plasma levels [11]. There were no other identifiable causes of the worsening of her ethanol withdrawal delirium. The chlordiazepoxide-associated delirium, confusion, and vital signs all appeared to improve initially with intramuscular lorazepam and subsequently with oral lorazepam. This case raises concern that the treatment approach of using a long-acting BNZ like chlordiazepoxide in nonelderly patients who meet criteria for ethanol withdrawal symptoms may not be fully benign and possibly a source of iatrogenic delirium. Iatrogenic delirium has been reported previously in a case series of four patients in the intensive care unit (ICU) setting as a complication of using BNZs for ethanol withdrawal [6], but occurred in patients with a higher risk of delirium due to other underlying pathologies, such as depression, malnutrition, or infection, head trauma, advanced age, or intubation, and without considering only objective symptoms [6, 8, 10]. However, in this case without predisposing factors and with use of only the patient’s vital signs (pulse and blood pressure) to drive the decision to administer chlordiazepoxide, the patient developed BNZ delirium as evidenced by the worsening of the delirium at anticipated peak chlordiazepoxide plasma levels.

Treatment of EWD is usually focused upon ameliorating agitation and other symptoms of neuronal hyperexcitability, which decreases the risk of seizures, injury, and mortality [12]. Long-acting BNZs, like chlordiazepoxide, are believed to be better owing to improvement with front-dosing [2], but do not appear to be superior in direct comparison studies with shorter half-life BNZs [9]. Additional preference for chlordiazepoxide is related to high lipophilicity, allowing for faster absorption through the oral than the intramuscular route, with peak plasma levels within 2 hours. [13–15]

While standard of care has benzodiazepines as a treatment of ethanol withdrawal, curiously, incapacitating benzodiazepine γ-aminobutyric acid (GABA) A receptors with the inverse agonist flumazenil may ameliorate symptoms of early ethanol withdrawal in some patients [15]. But the focus on GABA in EWD may be misplaced. Reductions in activity of the neurotransmitter acetylcholine have been hypothesized to play a role in delirium [16]. Several studies have suggested a potential anticholinergic effect of benzodiazepines alongside other anticholinergic medications in elderly patients with delirium. The risk for delirium in elders is greater when long-acting benzodiazepines [odds ratio (OR) 5.4, 95% confidence interval (CI) 1.0–29.2] or higher doses (OR 3.3, 95% CI 1.0–11.0) were given, compared with administration of short-acting benzodiazepines (OR 2.6, 95% CI 1.1–6.5) or lower doses (OR 2.6, 95% CI 0.8–9.1) [17]. However, chlordiazepoxide appears to have the weakest anticholinergic activity of seven examined benzodiazepines (bromazepam, camazepam, chlordiazepoxide, diazepam, lorazepam, medazepam, and triazolam), but any anticholinergic effect is approximately 1000-fold higher than achieved in human treatment [18]. Perhaps our patient had unknown predispositions to delirium that increased her risk when exposed to chlordiazepoxide.

## Conclusions

Recently, there has been increasing awareness of BNZ-related delirium in patients undergoing acute ethanol withdrawal. Our patient suggests that this may occur in individuals without typical predispositions for BNZ-induced delirium. While the current case is unlikely to alter the approach to the prevention and management of EWD, it is important for clinicians to be mindful that even standard-of-care medications are not always benign. Untoward worsening of delirium symptomatology with BNZs, particularly those with a long half-life, should lead one to consider the potential iatrogenic worsening of delirium. The mechanism by which chlordiazepoxide may have potentiated delirium in the setting of ethanol withdrawal is not clear.

## Data Availability

Not applicable.
